# Relationship between Biodistribution and Tracer Kinetics of ^11^C-Erlotinib, ^18^F-Afatinib and ^11^C-Osimertinib and Image Quality Evaluation Using Pharmacokinetic/Pharmacodynamic Analysis in Advanced Stage Non-Small Cell Lung Cancer Patients

**DOI:** 10.3390/diagnostics12040883

**Published:** 2022-04-01

**Authors:** Eveline Annette van de Stadt, Maqsood Yaqub, Robert C. Schuit, Imke H. Bartelink, Anke F. Leeuwerik, Lothar A. Schwarte, Adrianus J. de Langen, Harry Hendrikse, Idris Bahce

**Affiliations:** 1 Department of Pulmonology, Amsterdam UMC Location VUmc, 1081 HV Amsterdam, The Netherlands; i.bahce@amsterdamumc.nl; 2 Department of Radiology and Nuclear Medicine, Amsterdam UMC Location VUmc, 1081 HZ Amsterdam, The Netherlands; m.yaqub@amsterdamumc.nl (M.Y.); rc.schuit@amsterdamumc.nl (R.C.S.); n.h.hendrikse@amsterdamumc.nl (H.H.); 3 Department of Clinical Pharmacology and Pharmacy, Amsterdam UMC Location VUmc, 1081 HZ Amsterdam, The Netherlands; i.bartelink@amsterdamumc.nl (I.H.B.); a.f.leeuwerik@gmail.com (A.F.L.); 4 Department of Anesthesiology, Amsterdam UMC Location VUmc, 1081 HZ Amsterdam, The Netherlands; l.schwarte@amsterdamumc.nl; 5 Department of Thoracic Oncology, Netherlands Cancer Institute, 1066 CX Amsterdam, The Netherlands; j.d.langen@nki.nl

**Keywords:** non-small cell lung cancer, EGFR TKI PET/CT, biodistribution, ^11^C-erlotinib, ^18^F-afatinib, ^11^C-osimertinib

## Abstract

Background: Patients with non-small cell lung cancer (NSCLC) driven by activating epidermal growth factor receptor (EGFR) mutations are best treated with therapies targeting EGFR, i.e., tyrosine kinase inhibitors (TKI). Radiolabeled EGFR-TKI and PET have been investigated to study EGFR-TKI kinetics and its potential role as biomarker of response in NSCLC patients with EGFR mutations (EGFRm). In this study we aimed to compare the biodistribution and kinetics of three different EGFR-TKI, i.e., ^11^C-erlotinib, ^18^F-afatinib and ^11^C-osimertinib. Methods: Data of three prospective studies and 1 ongoing study were re-analysed; data from thirteen patients (EGFRm) were included for ^11^C-erlotinib, seven patients for ^18^F-afatinib (EGFRm and EGFR wild type) and four patients for ^11^C-osimertinib (EGFRm). From dynamic and static scans, SUV and tumor-to-blood (TBR) values were derived for tumor, lung, spleen, liver, vertebra and, if possible, brain tissue. AUC values were calculated using dynamic time-activity-curves. Parent fraction, plasma-to-blood ratio and SUV values were derived from arterial blood data. Tumor-to-lung contrast was calculated, as well as (background) noise to assess image quality. Results: ^11^C-osimertinib showed the highest SUV and TBR (AUC) values in nearly all tissues. Spleen uptake was notably high for ^11^C-osimertinib and to a lesser extent for ^18^F-afatinib. For EGFRm, ^11^C-erlotinib and ^18^F-afatinib demonstrated the highest tumor-to-lung contrast, compared to an inverse contrast observed for ^11^C-osimertinib. Tumor-to-lung contrast and spleen uptake of the three TKI ranked accordingly to the expected lysosomal sequestration. Conclusion: Comparison of biodistribution and tracer kinetics showed that ^11^C-erlotinib and ^18^F-afatinib demonstrated the highest tumor-to-background contrast in EGFRm positive tumors. Image quality, based on contrast and noise analysis, was superior for ^11^C-erlotinib and ^18^F-afatinib (EGFRm) scans compared to ^11^C-osimertinib and ^18^F-afatinib (EGFR wild type) scans.

## 1. Introduction

Non-small cell lung cancer (NSCLC) has one of the highest cancer-related mortalities worldwide [1,2,3]. Over the last decade, the treatment landscape for advanced stage NSCLC has been revolutionized by the development of targeted therapies against specific oncogenic driver pathways [4,5,6,7]. One of these drivers is the EGFR pathway. Wild-type EGFR is ligand-dependent, meaning it is only activated when its ligand is present. However, if an activating mutation is present in the ligand-binding domain of the receptor, activation occurs without the presence of a ligand, leading to unrestricted cell-growth and proliferation. EGFR TKI block this ligand-independent activation by binding to the kinase domain of the receptor with high affinity. Patients harboring an EGFR mutation positive tumor are best treated with an EGFR tyrosine kinase inhibitor (EGFR TKI), as tumor responses are higher and more durable with EGFR TKI than with chemotherapy or immunotherapy [8,9,10,11,12]. The currently available EGFR TKI can be categorized into three generations, varying in clinical efficacy and EGFR binding characteristics. Erlotinib, afatinib and osimertinib are examples of first-, second-, and third-generation EGFR TKI, respectively [11,13,14,15]. Currently, the preferred treatment for NSCLC patients harboring EGFR mutations is osimertinib, binding irreversibly to the mutated EGFR with high specificity [4,15].

Positron emission tomography (PET) using radiolabeled anticancer drugs provides a means to investigate the pharmacokinetic behavior of anticancer therapies in vivo [16]. The insights obtained with PET can be used for development of therapeutic strategies. Indeed, our group and others have shown that patients with EGFR mutated tumors can be identified using radiolabeled EGFR TKI as a PET tracer [17,18,19]. Also, high tumor uptake of ^11^C-erlotinib or ^18^F-afatinib was found to be associated with response to treatments using the respective TKI, highlighting the predictive value of EGFR TKI PET [16,20,21]. More recently, ^11^C-osimertinib has been developed as a novel PET tracer, for which the relationship between tumor uptake and tumor response to osimertinib is under investigation [22].

Assessing the biodistribution of these promising PET tracers can provide insight into their accumulation in tumor lesions and non-cancerous organs (off target binding). Comparing the biodistribution and the tracer kinetics of different generations of EGFR TKIs may be helpful to understand the differences seen in PET/CT image quality, but also, to understand drug behavior and predict target binding and receptor occupancy. The obtained knowledge may be used to predict the image quality of new tracers. However, to date, no biodistribution comparison analysis has been published on these EGFR directed PET tracers. Pharmacokinetic parameters of the used EGFR compounds may provide even further insight into the underlying mechanisms driving image quality.

We aimed to evaluate the biodistribution of ^11^C-erlotinib, ^18^F-afatinib and ^11^C-osimertinib, and compare their pharmacokinetic and pharmacodynamic behavior based on image analysis. We further aimed to summarize from literature various pharmacokinetic properties of the unlabeled compounds in relation to PET image quality.

## 2. Methods

### 2.1. Patient Inclusion

This is a retrospective study including patients that were enrolled in 3 previous and 1 ongoing EGFR TKI PET studies at our center. Patients were enrolled from the ^11^C-erlotinib cohort (dynamic and static), ^18^F-afatinib cohort (dynamic and static) and ^11^C-osimertinib (dynamic and static) ongoing study (CCMO number NL64722.031.18). Inclusion ranged from 2010–2019. Advanced stage NSCLC patients in whom PET/CT, EGFR mutational status and blood level measurements were available were selected. Since previous research has found that both common and uncommon mutations show similar tracer uptake, no distinction was made which activating EGFR mutation was present [17,21]. For ^18^F-afatinib, we included patients from 2 subgroups: those with EGFR mutated and those with EGFR wild type tumors. The ^11^C-erlotinib/^18^F-afatinib patients did not receive any prior EGFR TKI treatments, whereas the ^11^C-osimertinib patients were included after disease progression on a first-generation EGFR TKI (erlotinib or gefitinib; half-lifes of approximately 2 days). A two-week wash-out period was observed prior to scanning for each patient in the ^11^C-osimertinib study. All inclusion and exclusion criteria are provided in Supplement A.

### 2.2. Review Medical Ethics Committee

Each patient gave written informed consent prior to inclusion in their respective study. All studies were approved by the Medical Ethics Review Committee of either VU University Medical Center or the Netherlands Cancer Institute–Antoni van Leeuwenhoek (Amsterdam, The Netherlands).

### 2.3. Tracer Synthesis

^11^C-osimertinib was prepared by radiolabeling N-(5-((4-(1H-Indol-3-yl)pyrimidin-2-yl)amino)-2-((2-(dimethylamino)ethyl)(methyl)amino)-4-methoxyphenyl)acrylamide as a precursor with [^11^C]Methyl iodide ([^11^C]CH_3_I). After adding [^11^C]CH_3_I, it was distilled, dried over a NaOH/sicapent column and passed through a solution of osimertinib precursor in acetonitrile with NaH (60% dispersion in oil). This mixture is reacted at room temperature and diluted with water for injection. The crude product is purified by semi preparative HPLC after which the product is ready for formulation.

The tracer synthesis of ^11^C-erlotinib and ^18^F-afatinib was previously published by Bahce et al., and van de Stadt et al. [18,19].

### 2.4. PET/CT Scanning

All static ^11^C-erlotinib, ^18^F-afatinib and ^11^C-osimertinib emission scans were performed on an Ingenuity TF PET/CT scanner (Philips Medical Systems, Best, The Netherlands). ^11^C-erlotinib dynamic scans were performed on a Gemini TF PET/CT scanner (Philips Medical Systems, Best, The Netherlands). Both are high performance, time-of-flight (TOF), fully 3-dimensional PET scanners together with a 64 or 128-slice CT scanner. PET data were normalized and all appropriate corrections applied for dead time, decay, randoms, scatter and attenuation. A low dose CT scan (50 mAs, without iv or oral contrast) was performed for attenuation correction prior to each scan. ^11^C-osimertinib dynamic and static scans were performed on a single day, with a dynamic scan in the morning and a static scan in the afternoon to ensure radiation decay of the previous tracer injection.

#### 2.4.1. Dynamic Scanning

In the dynamic protocols, the injected dose of ^11^C-erlotinib was 370 MBq. This was followed by a dynamic PET scan with a duration of either 60 or 120 min. All dynamic PET emission scans were acquired in list-mode and reconstructed retrospectively using a 3-dimensional row-action maximum-likelihood algorithm into time frames with progressive increase in frame duration. For the 60-min dynamic emission scans, 36 frames were used: 1 × 10, 8 × 5, 4 × 10, 2 × 15, 3 × 20, 2 × 30, 6 × 60, 4 × 150, 4 × 300, and 2 × 600. For 120-min dynamic emission scans, 40 frames were used: 1 × 10, 8 × 5, 4 × 10, 2 × 15, 3 × 20, 2 × 30, 6 × 60, 4 × 150, 4 × 300, 2 × 600 and 4 × 900.

#### 2.4.2. Static Scanning

For ^11^C-erlotinib and ^11^C-osimertinib, 370 MBq was injected intravenously in the 40–70 min post-injection (p.i.) time interval. A static, whole-body scan was performed from the base of the skull (for ^11^C-erlotinib to the pelvis with 6 min per bed position, covering a total scan field of (up to) 81 cm. For ^18^F-afatinib, the same static, whole-body scan was performed directly following the 60-min dynamic emission scan (60–90 min p.i.). Reconstruction of PET data were performed using the BLOB-OS-TOF algorithm with CT based attenuation correction, resulting in a final voxel size of 4 × 4 × 4 mm and a spatial resolution of 5–7 mm full width at half maximum.

### 2.5. Blood Sampling & Metabolite Analysis

Both on-line and manual arterial sampling were performed during the dynamic PET scans. Manual samples were drawn to calibrate the on-line arterial curve, to determine plasma-to-whole blood ratios and to measure fractions of labelled metabolites in plasma using HPLC. On-line sampling was performed for 20–40 min p.i., 5 mL/min for the first 5 min and 1 mL/min for the remaining minutes. ^18^F-afatinib scans were sampled for 20 min, ^11^C-osimertinib scans for 40 min. Manual arterial samples were performed on 5, 10, 15, 30 and either 60 or 120 min p.i., depending on scan duration.

### 2.6. VOI Definition

All volumes of interest (VOIs) were defined manually by the same trained researcher using in-house developed software. Acquired CT images were used to define VOI definitions of tumors, with PET images projected in parallel, avoiding necrosis and blood vessels as much as possible. Healthy tissue was delineated using a fixed voxel size, depending on each organ.

### 2.7. Dynamic Scan SUV and TBR Analysis

Time-activity-curves (TACs) were derived from the tumor, blood pool, healthy lung tissue, vertebra, and if in field of view (FoV): liver and spleen VOIs. The TACs were subsequently corrected for patient weight and injected activity to obtain SUV curves using the following formula: SUV = C_image_ * W/IA, with C being the radioactivity in kBq/mL as measured using PET for each frame separately, BW bodyweight in kilogram and IA injected activity in MBq. Previous research of ^11^C-erlotinib and ^18^F-afatinib shows that tissue-to-blood ratios (TBR) are an adequate simplified measure to quantify tumor tracer uptake. Therefore, next, tissue-to-blood ratios (TBR) were calculated for each tissue VOI by dividing tissue SUV by blood pool SUV, calculated as described above.

### 2.8. Static Scan SUV and TBR Analysis

Tissue uptake was calculated for the following regions: brain (if in FoV), blood (descending aorta), healthy lung tissue (delineated in the contralateral lung), vertebra, liver, spleen, tumor and kidney. SUV and TBR values for each region were subsequently calculated using the same method as stated above.

Tumor contrasts relative to local healthy lung background were estimated using the formula: (SUV_tumor_/SUV_lung_ − 1) * 100% for SUV contrast, and (TBR_tumor_/TBR_lung_ − 1) * 100% for TBR. Noise (= coefficient of variation) in lung background was estimated using the formula: standard deviation/average * 100%.

### 2.9. Pharmacokinetic and Pharmacodynamic Interpretation

To improve understanding of the observed PET tracer behavior, a literature search was performed into the pharmacokinetic and pharmacodynamic (PKPD) parameters of the three EGFR TKI, using PubMED and the respective *Summary of Product Characteristics* (SmpCs). Labeling the TKI by substituting one carbon or fluorine atom as performed in this study did not change the molecular structure of the compound. Therefore, PKPD characteristics found for the non-labeled TKI could be extrapolated. If available, parameter predictions of micro vs. therapeutic PK were included. PKPD parameters of each TKI were summarized at the four levels of tumor drug penetration [23] (Supplement B Figure S1). First is the systemic level, represented by the volume of distribution, protein binding and rate of metabolism/clearance. The second level is the tissue level, which encompasses differences in diffusion and transport between tissues by receptors, lipophilicity and pKa of the compound. The third level entails the penetration of the drug at the cellular or molecular level in the target tissue. Parameters include affinity for the target receptor (represented by the dissociation constant (K_D_)) and lysosomal sequestration. Strong basic compounds like afatinib and osimertinib sequester in lysosomes since protonation will occur here to a greater extent than in the neutral environment of the cytosol. The protonated base is ‘trapped’ into the lysosomes due to reduced permeability [24]. The extend of lysosomal sequestration was calculated using a formula developed for basic lipophilic compounds [25]. Although lysosomes can be found in any cell (except red blood cells), the size and number of lysosomes in immune cells such as macrophages is larger [26]. Lung and spleen are rich in macrophages (lysosomal rich tissue), possibly leading to differences in uptake [27]. Lastly, the fourth level is the expression of pharmacological activity following target engagement. IC_50_ and AUC/IC_50_ is included in this step. IC_50_ resembles the drug concentration needed to achieve 50% inhibition of an enzyme. Since IC_50_ is dependent on drug exposure and these are highly different between the three TKIs at therapeutic dose levels, plasma area under the therapeutic concentration curve/IC_50_ (AUC/IC_50_) values were calculated. A ranked distribution pattern per tissue and tumor contrast relative to local healthy lung background by drug was then compared with the PKPD parameters. Ranking allowed to assess whether one or a combination of parameters could explain the differences in tracer uptake and image quality. Any parameter that ranked according to the image ranking per tissue and more specifically tumor-to-lung contrast was qualified as an important parameter to reflect PET/CT image quality.

### 2.10. Statistical Analysis

All PET analyses were performed using in-house developed software. Blood activity, parent fractions, SUV and TBR values are given with mean and standard deviation (SD) values. Area under the Curve (AUC) values of the TACs (60 min) are calculated using Excel and validated using SPSS version 26 (IBM, Armonk, NY, USA). Differences between ^18^F-afatinib AUC values of healthy tissues in EGFR wild type and EGFR mutated patients were tested using t-tests and were deemed significant if *p* ≥ 0.05.

## 3. Results

### 3.1. Baseline Characteristics

Twenty-four patients were included in this retrospective study. All subjects had an advanced stage NSCLC and mutational status was pathologically assessed prior to inclusion. Four scans were excluded due to technical failures (2 ^11^C-erlotinib scans in the static cohort and 2 static ^11^C-osimertinib scans). Three patients were excluded from the ^11^C-erlotinib dynamic cohort due to prior treatment with EGFR TKI. Three patients were excluded from the ^18^F-afatinib cohort due to scan protocol deviations. An overview of all included patients is provided in Table 1. A flowchart of included patients is provided in Supplement A. No adverse events or clinically detectable effects were observed in any of the patients with any of the tracers.

Thirteen ^11^C-erlotinib patients were included: five dynamic ^11^C-erlotinib scans and eight static ^11^C-erlotinib. Twelve tumors were identified. The average (±SD) injected dose was 387 ± 23 MBq. From the ^18^F-afatinib cohort, seven evaluable patients were derived. Four patients were wild-type EGFR, three harbored an EGFR mutation. Twelve tumors were identified. The average (±SD) injected dose was 350 ± 34 MBq. Four patients were included in the ongoing ^11^C-osimertinib study. An activating mutation was found in all four patients using molecular analysis of tumor DNA. In three patients the secondary resistance mutation T790M was found. Six scans were performed: three dynamic scans and three static scans. The average (±SD) injected dose was 323 ± 79 MBq. Six evaluable tumors were identified.

**Figure 1:**
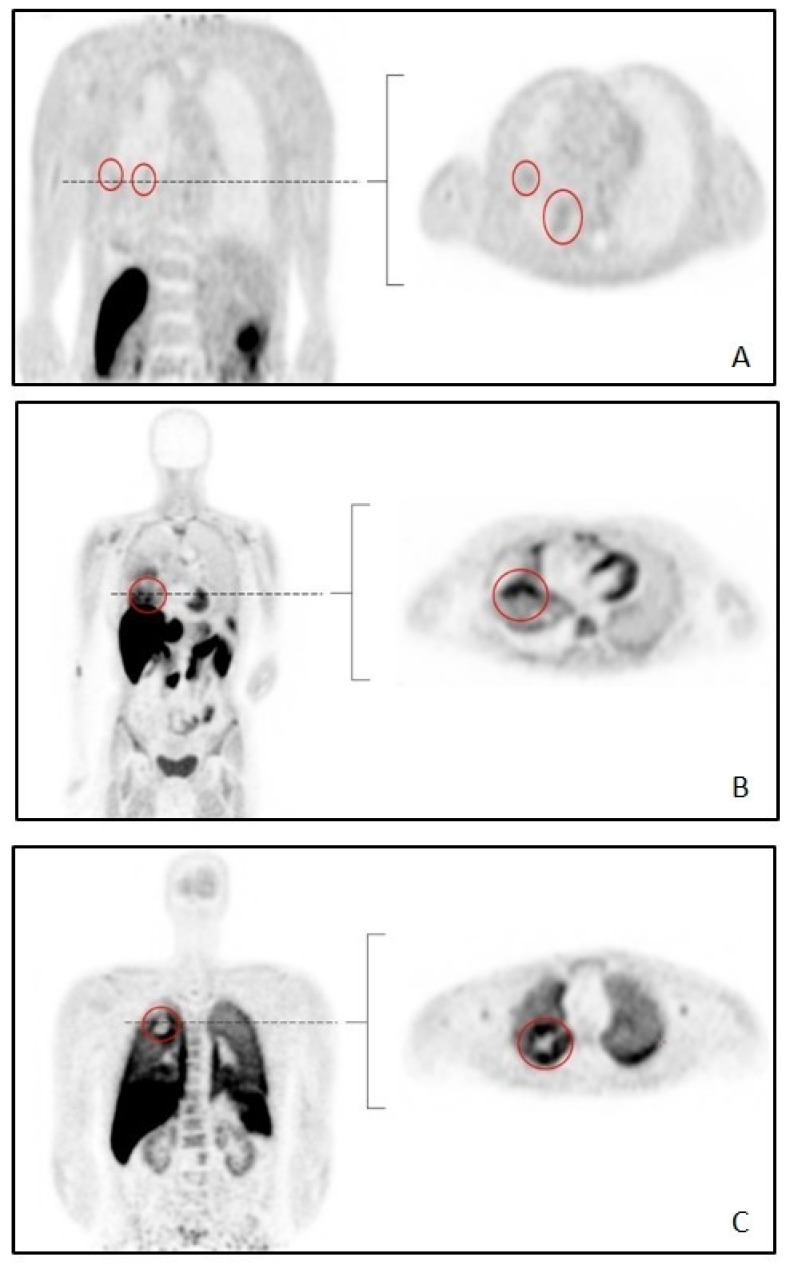
Typical examples of whole-body scans per tracer. Abbreviations: (**A**): 11C-erlotinib (**B**): 18F-afatinib (**C**): 11C-osimertinib. Tumors are circled in red.

All but one tumor from the combined cohort (^11^C-erlotinib, ^18^F-afatinib and ^11^C-osimertinib) were intrathoracically-located tumors. A pelvic metastasis found in patient 5 from the ^18^F-afatinib cohort was the single extrathoracic tumor included. Figure 1 shows typical examples of each tracer. 

### 3.2. Blood Activity Data

**Figure 2:**
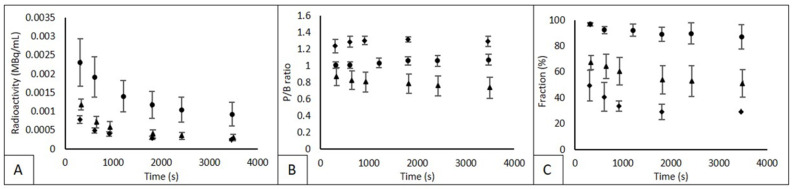
Whole blood radioactivity, P/B ratio and parent fraction. Abbreviations: Circles represent ^11^C-erlotinib, triangles represent ^18^F-afatinib and diamonds represent ^11^C-osimertinib in each graph. (**A**) whole-blood radioactivity, (**B**) plasma-to-whole-blood ratio (P/B). (**C**) the parent fraction. Mean and SD is given. In (**C**), the final measurement of 11c-osimertinib only 1 measurement could be used.

In Figure 2, mean whole-blood concentrations (corrected for injected dose and patient body weight), plasma-to-blood (P/B) ratios and parent fractions are shown for ^11^C-erlotinib, ^18^F-afatinib and ^11^C-osimertinib. For ^11^C-erlotinib, AUC of the whole-blood radioactivity concentration was 246 MBq/mL * s. For ^18^F-afatinib and ^11^C-osimertinib AUC values were 90 and 60 MBq/mL * s, respectively. This indicates a 3–4 times higher concentration in whole-blood for ^11^C-erlotinib during the total duration of the scan. AUC values for plasma activity were comparable, i.e., 258, 72 and 84 for ^11^C-erlotinib, ^18^F-afatinib and ^11^C-osimertinib, respectively. For ^11^C-erlotinib and ^11^C-osimertinib, plasma radioactivity is higher than whole-blood radioactivity, resulting in a median P/B ratio of 1.05 (±0.05) for ^11^C-erlotinib and 1.30 (±0.06) for ^11^C-osimertinib 60 min p.i. For ^18^F-afatinib, whole-blood radioactivity was higher resulting in a P/B ratio < 1 (median 0.79 ± 0.12). For ^11^C-erlotinib and ^11^C-osimertinib P/B ratios were relatively constant, but the P/B ratio for ^18^F-afatinib showed a slight decrease over the 60 min sampling period from 0.87 to 0.73.

^11^C-erlotinib parent fractions remained between 97% and 87%. ^18^F-afatinib parent fractions showed an initial rapid decline to 70% at 5 min post injection, followed by a gradual decrease to 35% at 60 min p.i. ^11^C-osimertinib parent fractions showed an even sharper initial decline with a drop to 50% and a decline further to a plateau of 28–29%, as shown in Figure 2.

### 3.3. Dynamic SUV and TBR Analysis

**Figure 3:**
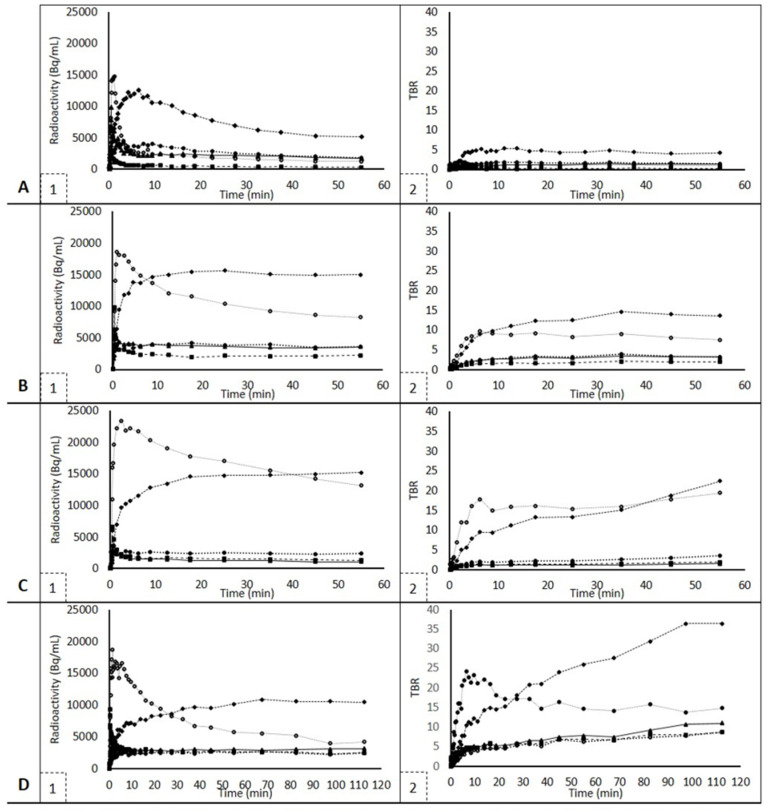
SUV and TBR TACs for each tracer. Figures on the left show SUV TACs, figures on the right show TBR TACs and are uniformly scaled apart from ^11^C-osimertinib scans, which were 120 minutes instead of 60 min. Figures (**A1**,**A2**) show ^11^C-erlotinib TACs. Figures (**B1**,**B2**) show ^18^F-afatinib TACs, EGFR mutated. Since these are typical examples, wild type ^18^F-afatinib TACs are shown in Figures (**C1**,**C2**). ^11^C-osimertinib TACs are shown in Figures (**D1**,**D2**). Squares represent lung, triangles represent tumor, diamonds represent liver, stroked circles (black line, white center) represent spleen and filled-in, black circles represent vertebra.

Typical TACs for SUV and TBR for lung and tumor tissue are shown in Figure 3. For dynamic scans, AUC values were calculated using the TACs of each tracer and each tissue, as shown in Table 2. Since the ^18^F-afatinib cohort consisted of both wild-type and EGFR mutated patients, both groups were analyzed separately, and, no significant difference in AUC values was found in all tissues except tumors (*p* = 0.01). Therefore, for all tissues except for the tumor, mean values for the entire ^18^F-afatinib cohort (EGFR wild type and EGFR mutated) are given.

For each tracer, uptake in the liver was highest. However, liver uptake of ^11^C-osimertinib was the highest, and ^11^C-erlotinib showed the lowest liver uptake. The spleen also showed high tracer uptake in all three tracers.

### 3.4. Static SUV and TBR Analysis

In Table 3, biodistribution results are given for both SUV and TBR. The blood SUV was highest in ^11^C-erlotinib (0.7 ± 0.1) and lowest in ^11^C-osimertinib (0.4, ±0.1). ^18^F-afatinib blood SUV was 0.5 (±0.1). For ^11^C-erlotinib, the tracer SUV and TBR uptake in spleen, kidney, tumor and vertebra were comparable. Lung tissue showed the lowest tracer uptake. ^18^F-afatinib showed comparable uptake values in wild type and EGFR mutated patients in non-target tissues. ^11^C-osimertinib showed the highest tracer uptake in lung and spleen tissue. Lung, kidney and vertebra show comparable uptake for all 3 TKIs. ^11^C-osimertinib showed higher brain uptake compared to ^18^F-afatinib, a finding which agrees with prior (pre)clinical biodistribution studies [28]. No brain tissue was scanned using ^11^C-erlotinib.

### 3.5. Tumor-to-Lung Contrast and Noise

Table 4 shows the contrast between tumor and lung tissue, and the background noise. To ensure contrast is a true difference between the 2 tissues and not background variability, noise was calculated for the lung tissue. For ^11^C-erlotinib and ^18^F-afatinib EGFR mutated groups, the contrast was larger than the noise, indicating that the difference between the two tissues is a true difference. Contrast was largest for ^11^C-erlotinib scans. In ^18^F-afatinib scans (EGFR mutated), contrast was sufficient to distinguish tumor and lung tissue, but lower than for ^11^C-erlotinib. Since ^11^C-osimertinib contrast values did not exceed the noise, the tumor uptake was inferior to the other two tracers. Therefore, image quality, as defined by the comparison of contrast to noise, was the highest for ^11^C-erlotinib, then ^18^F-afatinib, and then ^11^C-osimertinib.

### 3.6. Exploration of Tissue Distribution Based on Clinical PKPD Characteristics

PKPD parameters were stratified using the four levels of drug penetration and ranked according to obtained data. This ranking was then compared to PET and blood sample data. A summary is given in Table 5. All analyzed PKPD parameters are given in Supplement B, Table S1.

At the first level: ^11^C-erlotinib reached relatively high concentrations in the blood pool as illustrated by the (AUC) SUV in the aorta compared to ^18^F-afatinib and ^11^C-osimertinib (Table 2 and Table 3). This relates to a small (pharmacological) volume of distribution, similar to the volume of distribution at therapeutic dose levels (erlotinib 232 L, afatinib 2370 L and osimertinib 918 L, Table 5). However, the pharmacological volume of distribution did not correlate with tumor uptake and tumor-to-lung contrast.

The P/B ratio ranking observed in our study inversely correlates with the unbound drug fraction measured at therapeutic values: highest for osimertinib (at 1.66% unbound), median for erlotinib (at 8.8% unbound) and lowest for afatinib (9.48% unbound, appendix). However, the protein binding differences (ranked as: osimertinib < erlotinib < afatinib) did not rank according to the tumor uptake and contrast.

The observed liver uptake (ranked erlotinib < afatinib < osimertinib) did not rank according to the differences among metabolic intrinsic clearances of the tracers (Supplement B): The route of metabolism and hepatic extraction of the drug did not indicate a relationship with liver tissue nor tumor uptake and contrast.

At the second level: the tracer uptake ranking of ^18^F-afatinib being lower than ^11^C-osimertinib in brain tissues support the role of drug transporters. Drug efflux transporters play a major role in the blood-brain barrier, and therefore brain uptake of a drug. Afatinib has the highest affinity for drug efflux transporters BCRP and glycoprotein 1 (P-gP) and therefore the lower brain uptake compared to osimertinib may be the results of drug efflux by these transporters. Erlotinib has some affinity for these transporters. However, no ^11^C-erlotinib brain scans were performed. Drug transport did not rank according to tumor uptake and tumor-to-lung contrast.

At the third level: K_D_ is a measure of affinity for the target receptor. K_D_ values of erlotinib (2164), afatinib (2) and osimertinib (155) show a ranking that is not consistent with the ranking according to contrast data.

Spleen uptake of the three TKI ranked according to lysosomal sequestration, i.e., trapping of the drug in the lysosome. The lysosomal sequestration ranked according to lung-to-tumor and spleen contrast: erlotinib < afatinib < osimertinib.

At the fourth level: IC_50_ values are ranked erlotinib > osimertinib > afatinib, different from PET data. AUC/IC_50_ ratio ranked inversely to the PET data: erlotinib > afatinib > osimertinib.

## 4. Discussion

In this study, we assessed the biodistribution of three generations EGFR TKI PET tracers: ^11^C-erlotinib, ^18^F-afatinib and ^11^C-osimertinib using dynamic and static scans. We found differences in tracer pharmacokinetics and in the visual quality of the images which we quantified using tumor-to-lung contrast. Since labeling the TKI by substituting one carbon or fluorine atom does not change the molecular structure of the compound, PKPD parameters from unlabeled TKI were used to investigate the differences between tumor-to-lung contrast of these tracers.

The high uptake in liver and spleen in ^11^C-osimertinib and ^18^F-afatinib may have led to a sink effect, leading to a lower bioavailability of these tracers for target binding. For example, in ^11^C-osimertinib, parent fractions were very low when compared to the other two tracers, whereas the liver and spleen uptake was high. This effect was also seen for ^18^F-afatinib, although less pronounced. For ^11^C-erlotinib, however, parent fractions remained very high (>87%) throughout the entire duration of the scan, while liver uptake was not as high as the other two tracerc and especially spleen uptake was very low.

In absolute values, tumor tracer uptake seems lower for ^11^C-erlotinib than the other two tracers, however, images of the ^11^C-erlotinib scans of tumors in the lungs show a higher signal-to-noise ratio as compared to the other two tracers. This may be due to both contrast and noise, as a larger difference between target tissue uptake and surrounding tissue uptake could result in a better visibility. Furthermore, a higher tracer uptake variability of the surrounding tissue will make it harder to attribute any uptake differences to actual tissue differences. And, only contrast values exceeding the noise values for the surrounding tissue may be interpreted as an increased uptake signal. This is true for both ^11^C-erlotinib and ^18^F-afatinib, that show contrast values 3–4 times higher in EGFR mutation positive tumors as compared to noise values (Table 4). For ^11^C-osimertinib in EGFR mutations positive tumors and for ^18^F-afatinib in EGFR wild type tumor, contrast values stay within the noise range. For ^11^C-osimertinib, contrast was even shown to be negative, meaning the background tissue of the lung showed higher tracer uptake than the tumor itself. This results in less pronounced images, even though in absolute numbers, tumor uptake (SUV, TBR) would seem adequate.

Our exploratory results of the whole-body drug distribution based on PKPD characteristics suggests that multiple PKPD parameters related to physicochemical drug properties such as protein binding; protonation in the acidic environment of the lysosome, resulting in lysosomal sequestration; and target binding differences potentially have the highest impact on predicting microdose biodistribution and PET image quality. Particularly the high uptake of ^11^C-osimertinib and (very) low uptake of ^11^C-erlotinib in lysosomal-rich tissue such as the spleen and (non-tumorous) lung tissue seems to indicate the importance of lysosomal sequestration. Lysosomal sequestration has also shown to play an important role in resistance to several EGFR TKI [34,35]. By combining these processes in a physiologically-based pharmacokinetic model, more information could be obtained. Physiologically-based pharmacokinetic (PBPK) models are mathematical representations of tissues and organs, where each of the compartments correspond with physiological volumes of the tissues and organs included [36]. These models can be used to assess the contribution of each of the identified processes on whole body TKI-distribution. PBPK modeling may help to predict differences in tissue uptake and predict image quality of (new) EGFR tracer TKIs. Using this information, derived from TKI-PET, may help in the development of new drugs, e.g., by predicting whether the drug reaches its target, thereby reducing the risks to humans by limiting drug exposure during first-in-human dose-finding trials, and reducing the associated research time and costs, involved with drug manufacturing requirements [37].

## 5. Conclusions

Tracer kinetic and biodistribution comparison of ^11^C-erlotinib, ^18^F-afatinib and ^11^C-osimertinib showed that ^11^C-erlotinib and ^18^F-afatinib had the highest tumor-to-lung contrast in EGFR mutated tumors. ^11^C-erlotinib showed the least background noise. Using contrast and noise, quality of the images of the ^11^C-erlotinib and ^18^F-afatinib (EGFR mutated) scans were superior to the ^11^C-osimertinib and ^18^F-afatinib (EGFR wild type) scans. Spleen uptake was notably high in ^18^F-afatinib and ^11^C-osimertinib patients, possibly explaining their lower tracer availability in blood as compared to ^11^C-erlotinib. Lysosomal accumulation in immune-rich tissue may explain the high spleen uptake in both tracers. PBPK modeling taking into account aspects such as protein binding, lysosomal sequestration and targetbinding may help improve our understanding of the EGFR TKI tracer behavior at microdose (tracer) level and predict image quality of (future) EGFR tracers.

## 6. Limitations

Technical difficulties in measuring parent fractions at lower ranges, potentially related to the short half-life of ^11^C-osimertinib, sample instability and adduct formation may have affected the blood sample analysis of ^11^C-osimertinib blood samples [38]. The data obtained after 20 min. p.i. may be considered less accurate, hindering the creation of reliable input functions, necessary to perform pharmacokinetic modeling [39]. Consequently, no preferred uptake parameter for ^11^C-osimertinib could be proposed.

Furthermore, for ^11^C-erlotinib two cohorts were used: one for the dynamic scan, and one for the static, whole-body scan, as no cohort was available in which both scan types were performed in the same patient. However, results from dynamic or static scans show comparable results. For the other tracers, all patients underwent both scan types.

Another limitation is the uncertain predictive value of the biodistribution of micro-dosed tracers to reflect therapeutic PK. It is possible that for these three EGFR TKIs differences in pharmacokinetic and pharmacodynamic properties known at a therapeutic level are not appropriate to explain the differences in tumor-to-lung contrast and PET image quality. PKPD properties can be different using microdoses due to saturable processes such as receptor abundance [37]. Modeling and simulation tools may help to address this.

## 7. Future Consideration

As mentioned above, future research should focus on pharmacokinetic modeling to obtain the preferred uptake parameter for ^11^C-osimertinib. In our research, low parent fractions were the reason we could not perform this analysis. Furthermore, PBPK modeling and simulation could help improve our understanding of the EGFR TKI tracer behavior at microdose (tracer) level.

## Figures and Tables

**Table 1:** Baseline characteristics.

Study *	EGFR TKI Tracer	Patient Nr	Age	Gender ^@^	EGFR Mutation ^&^	Prior TKI	Dynamic Scan	Static Scan
1	^11^C-erlotinib	1	80	M	Yes	No	Yes	--
		2	73	M	Yes	No	Yes	--
		3	55	F	Yes	No	Yes	--
		4	80	F	Yes	No	Yes	--
		5	82	F	Yes	No	Yes	--
2	^11^C-erlotinib	1	69	M	Yes	No	--	Yes
		2	59	M	Yes	No	--	Yes
		3	52	F	Yes	No	--	Yes
		4	67	F	Yes	No	--	Yes
		5	70	F	Yes	No	--	Yes
		6	83	M	Yes	No	--	Yes
		7	83	M	Yes	No	--	Yes
		8	54	M	Yes	No	--	Yes
3	^18^F-afatinib	1	53	F	Yes	No	Yes	Yes
		2	69	M	No	No	Yes	Yes
		3	71	M	No	No	Yes	Yes
		4	47	F	Yes	No	Yes	Yes
		5	71	F	Yes	No	Yes	Yes
		6	78	M	No	No	Yes	Yes
		7	54	M	No	No	Yes	Yes
4	^11^C-osimertinib	1	58	F	Yes	Yes ^#^	Yes	Yes
		2	81	M	Yes	Yes ^#^	Yes	No
		3	77	F	Yes	Yes ^#^	No	Yes
		4	43	M	Yes	Yes ^#^	Yes	Yes

**Comments:** (*) protocols are described in the methods section. (**^@^**) M are male patients, F female. (**^#^**) patients 1, 2 and 4 were treated with erlotinib prior to scanning. Patient 3 was treated with gefitinib prior to scanning. A wash-out period of 14 days preceded scanning of all ^11^C-osimertinib patients. (**^&^**) EGFR mutations are activating mutations.

**Table 2:** AUC values.

		^11^C-Erlotinib	^18^F-Afatinib		^11^C-Osimertinib
SUV-AUC	Spleen	110.1 (25.0)	560.3 (76.7)		757.1 (340.8)
	Liver	518.3 (228.6)	731.5 (87.4)		679.1 (256.0)
	Vertebra	119.8 (20.4)	154.3 (54.5)		171.6 (67.4)
	Tumor	85.6 (28.3)	*EGFR* +: *Wild type*:	244.8 (65.1) 105.4 (25.9)	192.9 (116.9)
	Lung	24.7 (8.2)	98.5 (16.5)		243.1 (112.5)
	Aorta	95.4 (21.9)	80.6 (25.9)		50.5 (18.7)
TBR-AUC	Spleen	62.9 (4.5)	531.4 (281.7)		982.7 (55.4)
	Liver	460.1 (305.1)	747.7 (426.3)		1252.0 (109.3)
	Vertebra	85.3 (11.2)	143.4 (57.6)		268.0 (58.0)
	Tumor	61.6 (23.5)	*EGFR* +: *Wild type*:	150.7 (9.1) 113.0 (44.8)	279.9 (109.3)
	Lung	16.0 (5.8)	90.9 (37.9)		361.0 (137.2)

**Comments:** SUV and TBR values are given per tracer for each tissue. Mean values are given with standard deviations in brackets. For ^18^F-afatinib, values for EGFR mutated and wild type tumors are given separately. SUV values are calculated using MBq/mL and seconds, TBR values are calculated using TBR and seconds.

**Table 3:** Static SUV and TBR values given per tracer.

		^11^C-Erlotinib	^18^F-Afatinib		^11^C-Osimertinib
SUV	Spleen	1.0 (0.4)	6.8 (1.0)		6.8 (1.5)
	Liver	7.6 (4.0)	11.8 (2.2)		13.5 (3.6)
	Vertebra	0.8 (0.3)	2.4 (0.8)		1.6 (0.4)
	Tumor	0.9 (0.3)	*EGFR* +: *Wild type*:	1.8 (1.3) 1.6 (0.1)	2.1 (0.9)
	Lung	0.3 (0.1)	1.3 (0.5)		2.6 (0.8)
	Kidney	1.1 (0.4)	3.5 (0.7)		2.0 (0.3)
	Brain	N/A	0.04 (0.01)		0.3 (0.1)
	Aorta	0.7 (0.3)	0.5 (0.1)		0.4 (0.1)
TBR	Spleen	1.5 (0.4)	13.2 (2.3)		12.8 (7.7)
	Liver	12.3 (6.9)	23.3 (6.2)		36.0 (6.7)
	Vertebra	1.2 (0.2)	4.8 (2.0)		4.2 (0.7)
	Tumor	1.4 (0.5)	*EGFR* +: *Wild type*:	3.6 (2.4) 3.3 (1.0)	5.6 (2.0)
	Lung	0.5 (0.2)	2.5 (1.2)		7.0 (1.6)
	Kidney	1.7 (0.6)	6.9 (1.8)		5.6 (1.2)
	Brain	N/A	0.08 (0.03)		0.8 (0.5)

**Comments:** SUV and TBR values are given per tracer for each tissue. Mean values are given and standard deviations are in brackets.

**Table 4:** Tumor-to-Lung Contrast and Noise Values.

	Contrast *SUV (%)*	Contrast *TBR (%)*	Noise *SUV (%)*	Noise *TBR (%)*
**^11^C-erlotinib**	167.0	178	42.5	29.5
**^18^F-afatinib** *wild type*	15.5	20.5	44.3	50.8
**^18^F-afatinib** *EGFR mutated*	96	95.9	33.6	11.4
**^11^C-osimertinib**	−19.8	−20.5	30.4	22.9

**Table 5:** PKPD parameters.

		Erlotinib	Afatinib	Osimertinib	References
Level					
**1**	*Volume of distribution (L)*	232	2370	918	[29,30,31]
	*Fraction unbound (%)*	8.77	9.48	1.66	[28]
**2**	*Drug transport (efflux ratio MDR1 (P-gp)/BRCP)*	6.9	53.1	3.2	[28]
	*Strength of basicity (pKa)*	Weak–5.5	Strong–8.2	Strong–9.0	[28]
**3**	*Kd EGFR (nM)*	2164	2	155	[32]
	*Lysosomal sequestration (%)*	<1	53.15	54.32	[25]
**4**	*IC50 exon19del (nM)*	7	0.8	17	[33]
	AUC/IC50 (nM * h/L)	11260	2336	702	--

## Data Availability

Datasets used in this study are available upon reasonable request from the corresponding author.

## Supplementary Materials

The following supporting information can be downloaded at: https://www.mdpi.com/article/10.3390/diagnostics12040883/s1, Supplement A: Figure S1: Total screened patients are depicted in the top row; Supplement B: Figure S2: The pathway of drug administration to the tumor response is affected by tumor drug penetration at four levels; Table S1: TKI parameters at standard daily dose level related to systemic level (absorption, distribution, metabolism and excretion), the tumor tissue level and the cellular/molecular level and pharmacological level; References [40,41,42,43,44,45,46,47,48] are cited in the supplementary materials.
