# Supplementary material for: Relationship between Biodistribution and Tracer Kinetics of 11C-Erlotinib, 18F-Afatinib and 11C-Osimertinib and Image Quality Evaluation Using Pharmacokinetic/Pharmacodynamic Analysis in Advanced Stage Non-Small Cell Lung Cancer Patients
Source: Diagnostics (Basel). 2022 Apr 1;12(4):883. doi: 10.3390/diagnostics12040883 (PMC9032381; doi:10.3390/diagnostics12040883)
Supplement: Supplementary file 1 [file diagnostics-12-00883-s001.zip › diagnostics-1573032-supplementary.pdf]

Supplement A:

Flowchart patient inclusion

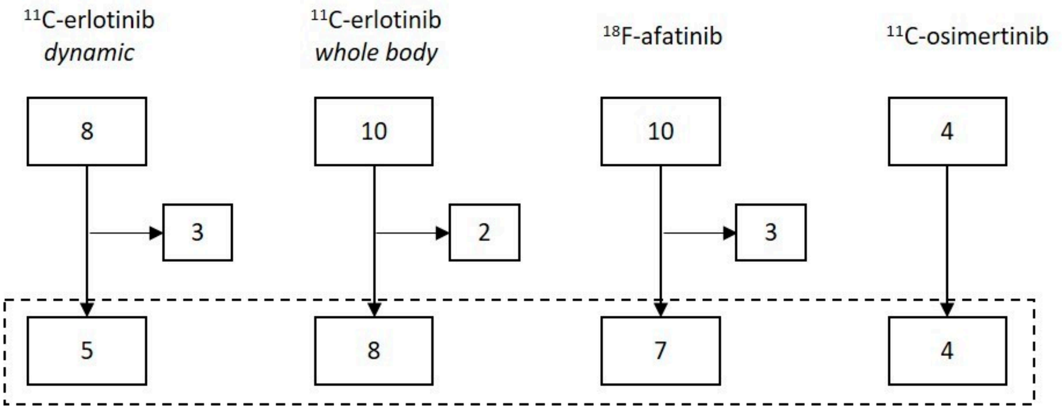

Figure S1. Total screened patients are depicted in the top row. Included patients are shown in the bottom row and circled by the dotted line. Excluded patients are depicted in the middle row. For erlotinib: Five patients were derived from the dynamic 11C-erlotinib cohort comprised of 8 patients total. Six evaluable tumors were identified. Injected dose was 365 ± 14 MBq. Ten patients were included in the static 11C-erlotinib study. An activating mutation was found in all patients using molecular analysis of tumor DNA. In 2 patients, due to technical failures the scans were not evaluable. In the 11C-osimertinib group, no patients were excluded because in each patient at least 1 scan was usable. Production of 11C-osimertinib failed once, resulting in one patient only undergoing a dynamic scan (patient 2). Technical failure of one dynamic scan resulted in a patient where only the whole-body scan was evaluable (patient 3).

In- and exclusion criteria 11C-osimertinib PET/CT
Inclusion criteria

1. Have a histologically or cytologically confirmed diagnosis of stage IV NSCLC, characterized by a sensitizing EGFR mutation
2. Have progressive disease according to RECIST 1.1 on a first or second generation EGFR TKI and still receiving the drug.
3. Provision of informed consent prior to any study specific procedures
4. Patients must be > 18 years of age.
5. World Health Organization (WHO) performance status 0-2.
6. Patients must have a life expectancy ⩾ 12 weeks.
7. Females should be using adequate contraceptive measures, should not be breast feeding and must have a negative pregnancy test prior to start of dosing if of child-bearing potential or must have evidence of non-child-bearing potential by fulfilling one of the following criteria at screening:
• Post-menopausal defined as aged more than 50 years and amenorrheic for at least 12 months following cessation of all exogenous hormonal treatments

• Women under 50 years old would be consider postmenopausal if they have been amenorrheic for 12 months or more following cessation of exogenous hormonal treatments and with LH and FSH levels in the post-menopausal range for the institution
• Documentation of irreversible surgical sterilisation by hysterectomy, bilateral oophorectomy or bilateral salpingectomy but not tubal ligation
8. Male patients should be willing to use barrier contraception (see Restrictions, Section 3.6).
9. At least one lesion, not previously irradiated, that can be accurately measured at baseline as ⩾ 10 mm in the longest diameter (except lymph nodes which must have short axis ⩾ 15 mm) with computed tomography (CT) or magnetic resonance imaging (MRI) and which is suitable for accurate repeated measurements.
Exclusion criteria

1. Involvement in the planning and/or conduct of the study (applies to both sponsor staff and/or staff at the study site)
2. Previous treatment with osimertinib
3. Treatment with an investigational drug within five half-lives of the compound
4. Patients currently receiving (or unable to stop) medications or herbal supplements known to be potent inhibitors of CYP3A4 (at least 1 week prior) and potent inducers of CYP3A4 (at least 3 week prior).
5. Any unresolved toxicities from prior therapy greater than CTC AE grade 1 at the time of study.
6. Any evidence of severe or uncontrolled systemic diseases, including uncontrolled hypertension and active bleeding diatheses, or active infection including hepatitis B, hepatitis C and human immunodeficiency virus (HIV).
7. Patients with symptomatic CNS metastases who are neurologically unstable
8. Past medical history of ILD, drug-induced ILD, radiation pneumonitis requiring steroid treatment, or any evidence of clinically active ILD.
9. Inadequate bone marrow reserve or organ function as demonstrated by any of the following laboratory values:
• Absolute neutrophil count <1.5 × 109/L.
• Platelet count <100 x 109/L.
• Haemoglobin <90 g/L.
• Alanine aminotransferase >2.5 times the upper limit of normal (ULN) if no demonstrable liver metastases or >5 times ULN in the presence of liver metastases.
• Aspartate aminotransferase >2.5 times ULN if no demonstrable liver metastases or >5 times ULN in the presence of liver metastases.
• Total bilirubin >1.5 times ULN if no liver metastases or >3 times ULN in the presence of documented Gilbert's Syndrome (unconjugated hyperbilirubinaemia) or liver metastases.
• Creatinine >1.5 times ULN concurrent with creatinine clearance <50 ml/min (measured or calculated by Cockcroft and Gault equation); confirmation of creatinine clearance is only required when creatinine is >1.5 times ULN.
10. Any of the following cardiac criteria:
• Mean resting corrected QT interval (QTc using Fredericia's formula) >470 mSec.

• Any clinically important abnormalities in rhythm, conduction or morphology of resting ECG (e.g., complete left bundle branch block, third degree heart block, second degree heart block).
• Any factors that increase the risk of QTc prolongation or risk of arrhythmic events such as heart failure, hypokalemia, congenital long QT syndrome
11. History of hypersensitivity to osimertinib (or drugs with a similar chemical structure or class to osimertinib) or any excipients of these agents.
12. Males and females of reproductive potential who are not using an effective method of birth control and females who are pregnant or breastfeeding or have a positive (urine or serum) pregnancy test prior to study entry.

Supplement B:

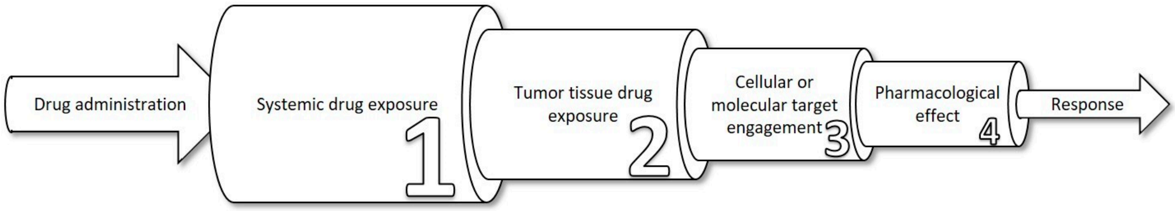

Figure S2. The pathway of drug administration to the tumor response is affected by tumor drug penetration at four levels: (1) the systemic level (the concentration of the drug in the blood pool, which determines how much of the drug is available for tumor penetration), (2) the tissue level (e.g., is the drug able to distribute throughout the tumor tissue, as influenced by the tumor microenvironment), (3) the cellular or molecular engagement level (where the drug is able to engage and interact with its target at the cellular/molecular level, a proximal or direct measure of drug mechanism of action), and (4) the expression of pharmacological activity following target engagement (a distal or indirect measure of drug pharmacodynamics). All these levels will be affected by responses to treatment.

Table S1. TKI parameters at standard daily dose level related to systemic level (absorption, distribution, metabolism and excretion), the tumor tissue level and the cellular/molecular level and pharmacological level

**Level**			**Erlotinib**	**Afatinib**	**Osimertinib**	ref
**1. Systemic**	**Absorption**	Ka *(h^{-1})*	0.949	0.252	0.24	(1-3)
		Bioavailability *(%)*	59	92	70	(4-6)
		Time to Css *(days)*	7-8	8	15	(2, 3, 7)
		C_{max} at Css *(nM)*	4651	101	501	(1, 3, 7)
		C_{min} at Css *(nM)*	2567	N/A	417	[1,3]
		Target C_{trough} *(nM)*	1271	50	332	(8-10)
		AUC at Css *(µM*h)*	79	2	12	(2, 11, 12)

		AUC dose normalized *(µM*h)*	207	23	75	(2, 11, 12)
	**Distribution**	Unbound fraction plasma *(%)*	8.77	9.48	1.66	(9)
		Vd *(L)*	232	2370	918	(2, 3, 11)
	**Metabolism**	Liver *(%)*	±98	N/A	±99	(11, 13, 14)
		Enzymes involved	Main: CYP3A4 Also: CYP1A1/2 Strong UGT1A1 and CYP1A1 inhibitor	N/A	Oxydation CYP3A4 Dealkylation CYP3A5	(3, 4, 11)
		Active metabolites	OSI-420, OSI413 (10% AUC)	None	AZ7550 (10% AUC, potency as OSI) AZ5104 (10% AUC, higher potency)	(3, 4, 11)
		Extrahepatic	Lung (CYP1A1) GI-tract (CYP3A4) Tumor (CYP1B1)	Major metabolites consist of covalent adducts to proteins	Potential extrahepatic conjugation, covalent protein binding	(3, 4, 11)
	**Elimination**	$T_{1/2}$ *(h)*	36.2	45	48.6	(3-5)
		Cl *(L/h/F)*	3.95	44	14.2	(1, 3, 11)
		Renal extraction *(%)*	9, < 2 unchanged	4.29	14.2, 2 unchanged	(4, 11, 14)
**2. (tumor) tissue**		Efflux ratio MDR1 (P-gp)/BRCP	6.9	53.1	3.2	(9)
		Log P	3.3	3.6	3.2	(9)
**3. Cellular - molecular**		Kd EGFR (nM)	2164	2	155	(15)
		Lysosomal trapping *(%)****	<1	53.15	54.32	(16)
**4. Pharmacology**		IC50 ex19del (nM)	7	0.8	17	(17)
		IC50 ex70ins (nM)	1273.6	6.1	OSI: 96.6 AZ5104: 9.5	(17)
		IC50 T790M (nM)	1197	56.7	OSI: 1 AZ5104: <1 AZ7550: 0	(17)
		IC50 T790WT (nM)	1020	31	OSI: 184 AZ5104: 25 AZ7550: 519	(17)
		AUC/IC50 (nM*h/L)	11260	2336	702	

*** Schmitt *et al.* was used to predict the lysosomal accumulation for the two lipophilic basic compounds. Lipophilicity and pKA may play a role in this (ref) and is common for drugs that are susceptible to protonation at the physiological pH range. Once the drug enters the acidic compartments, such as the acidic lysosomes (cytoplasm pH ±7.2 versus lysosome pH ±5), bases with a pKa>6 will be protonated. This protonated form cannot easily diffuse back into the cytosol. As a result, high concentrations of the compound can accumulate in the lysosome. Afatinib and osimertinib are diprotic bases (afatinib pKa1, 5.1; pKa2, 8.2, pKa1, 4.7; pKa2, 9.0) erlotinib has a pKa <6 (ref). Formula does not apply to erlotinib which is a basic compound with pKa values <7. For such compounds no substantial impact of phospholipid binding or lysosomal sequestration is expected.

Css = steady state

C_{max} = maximum concentration

C_{min} = minimum concentration

C_{trough} = lowest concentration before administration of the next scheduled dose

AUC = area under the therapeutic concentration curve

Vd = volume of distribution

$t_{1/2}$ = Half-life, time needed to achieve half the concentration from C_{max}

CL= clearance

Log P = measure of lipophilicity

K_D = the dissociation constant and describes the concentration needed to occupy 50% of receptors. In other words: a low K_D means less TKI molecules are needed to occupy 50% of the EGF receptors, indicating a higher affinity. A higher affinity as seen in EGFR mutated tumors versus wild-type tumors is correlated with higher tumor tracer uptake in ^{11}C-erlotinib and ^{18}F-afatinib(18, 19).

IC50 = concentration needed to achieve 50% inhibition of the target enzyme

Supplement

1. Lu, J.F.; Eppler, S.M.; Wolf, J.; Hamilton, M.; Rakhit, A.; Bruno, R.; Lum, B.L. Clinical pharmacokinetics of erlotinib in patients with solid tumors and exposure-safety relationship in patients with non-small cell lung cancer. *Clin. Pharmacol. Ther.* **2006**, *80*, 136–145.
2. Katsuya, Y.; Fujiwara, Y.; Sunami, K.; Utsumi, H.; Goto, Y.; Kanda, S.; Horinouchi, H.; Nokihara, H.; Yamamoto, N.; Takashima, Y.; et al. Comparison of the pharmacokinetics of erlotinib administered in complete fasting and 2 h after a meal in patients with lung cancer. *Cancer Chemother. Pharmacol.* **2015**, *76*, 125–132.
3. Brown, K.; Comisar, C.; Witjes, H.; Maringwa, J.; de Greef, R.; Vishwanathan, K.; Cantarini, M.; Cox, E. Population pharmacokinetics and exposure-response of osimertinib in patients with non-small cell lung cancer. *Br. J. Clin. Pharmacol.* **2017**, *83*, 1216–1226.
4. (EMA) EMA. Tarceva (INN-erlotinib) Summary of Product Characteristics 2005. Available online: https://www.ema.europa.eu/en/documents/product-information/tarceva-epar-product-information_en.pdf (accessed on 10 January 2022).
5. Medicines CfMPfHUCE. Giotrif (INN-Afatinib) European Public Assessment Report 2013. Available online: https://www.ema.europa.eu/en/documents/assessment-report/giotrif-epar-public-assessment-report_en.pdf (accessed on 10 January 2022).
6. Medicines. CfMPfHUCE. Tagrisso (INN-Osimertinib) European Public Assessment Report 2015. Available online: http://www.ema.europa.eu/en/documents/assessment-report/tagrisso-epar-public-assessment-report_en.pdf (accessed on 10 January 2022).
7. Nakao, K.; Kobuchi, S.; Marutani, S.; Iwazaki, A.; Tamiya, A.; Isa, S.; Okishio, K.; Kanazu, M.; Tamiya, M.; Hirashima, T.; et al. Population pharmacokinetics of afatinib and exposure-safety relationships in Japanese patients with EGFR mutation-positive non-small cell lung cancer. *Sci. Rep.* **2019**, *9*, 18202.
8. Yu, H.; Steeghs, N.; Nijenhuis, C.M.; Schellens, J.H.; Beijnen, J.H.; Huitema, A.D. Practical guidelines for therapeutic drug monitoring of anticancer tyrosine kinase inhibitors: Focus on the pharmacokinetic targets. *Clin. Pharmacokinet.* **2014**, *53*, 305–325.
9. Colclough, N.; Chen, K.; Johnstrom, P.; Strittmatter, N.; Yan, Y.; Wrigley, G.L.; Schou, M.; Goodwin, R.J.; Varnäs, K.; Adua, S.J.; et al. Preclinical Comparison of the Blood-brain barrier Permeability of Osimertinib with Other EGFR TKIs. *Clin. Cancer Res.* **2021**, *27*, 189–201.
10. Medicines CfMPfHUCE. Osimertinib European Public Assessment Report 2015. Available online: http://www.ema.europa.eu/docs/en_GB/document_library/EPAR_Public_assessment_report/human/004124/WC500202024.pdf (accessed on 10 January 2022).

11. Wind, S.; Schnell, D.; Ebner, T.; Freiwald, M.; Stopfer, P. Clinical Pharmacokinetics and Pharmacodynamics of Afatinib. *Clin. Pharmacokinet*. **2017**, *56*, 235–250.
12. Research FaDACfDEa. Osimertinib Clinical Pharmacology and Biopharmaceutics Review 2015. Available online: https://www.accessdata.fda.gov/drugsatfda_docs/nda/2015/208065orig1s000clinpharmr.pdf (accessed on 10 January 2022).
13. Ling, J.; Johnson, K.A.; Miao, Z.; Rakhit, A.; Pantze, M.P.; Hamilton, M.; Lum, B.L.; Prakash, C. Metabolism and excretion of erlotinib, a small molecule inhibitor of epidermal growth factor receptor tyrosine kinase, in healthy male volunteers. *Drug Metab. Dispos.* **2006**, *34*, 420–426.
14. Dickinson, P.A.; Cantarini, M.V.; Collier, J.; Frewer, P.; Martin, S.; Pickup, K.; Ballard, P. Metabolic Disposition of Osimertinib in Rats, Dogs, and Humans: Insights into a Drug Designed to Bind Covalently to a Cysteine Residue of Epidermal Growth Factor Receptor. *Drug Metab. Dispos.* **2016**, *44*, 1201–1212.
15. Joly-Tonetti, N.; Ondet, T.; Monshouwer, M.; Stamatas, G.N. EGFR inhibitors switch keratinocytes from a proliferative to a differentiative phenotype affecting epidermal development and barrier function. *BMC Cancer* **2021**, *21*, 5.
16. Schmitt, M.V.; Lienau, P.; Fricker, G.; Reichel, A. Quantitation of Lysosomal Trapping of Basic Lipophilic Compounds Using In Vitro Assays and In Silico Predictions Based on the Determination of the Full pH Profile of the Endo-/Lysosomal System in Rat Hepatocytes. *Drug Metab. Dispos.* **2019**, *47*, 49–57.
17. Hirano, T.; Yasuda, H.; Tani, T.; Hamamoto, J.; Oashi, A.; Ishioka, K.; Arai, D.; Nukaga, S.; Miyawaki, M.; Kawada, I.; et al. In vitro modeling to determine mutation specificity of EGFR tyrosine kinase inhibitors against clinically relevant EGFR mutants in non-small-cell lung cancer. *Oncotarget* **2015**, *6*, 38789–38803.
18. Bahce, I.; Smit, E.F.; Lubberink, M.; van der Veldt, A.A.; Yaqub, M.; Windhorst, A.D.; Schuit, R.C.; Thunnissen, E.; Heideman, D.A.M.; Postmus, P.E.; et al. Development of [(11)C]erlotinib positron emission tomography for in vivo evaluation of EGF receptor mutational status. *Clin. Cancer Res.* **2013**, *19*, 183–193.
19. Van de Stadt, E.A.; Yaqub, M.; Lammertsma, A.A.; Poot, A.J.; Schuit, R.C.; Remmelzwaal, S.; Schwarte, L.A.; Smit, E.F.; Hendrikse, H.; Bahce, I.. Identifying advanced stage NSCLC patients who benefit from afatinib therapy using (18)F-afatinib PET/CT imaging. *Lung Cancer* **2021**, *155*, 156–162.
